# Semi-Automated Control System for Reaching Movements in EMG Shoulder Disarticulation Prosthesis Based on Mixed Reality Device

**DOI:** 10.1109/OJEMB.2021.3058036

**Published:** 2021-02-09

**Authors:** Shunta Togo, Kazuaki Matsumoto, Susumu Kimizuka, Yinlai Jiang, Hiroshi Yokoi

**Affiliations:** Graduate School of Informatics and EngineeringThe University of Electro-Communications Tokyo 1828585 Japan; Center for Neuroscience and Biomedical EngineeringThe University of Electro-Communications 13133 Tokyo 1828585 Japan; Graduate School of Informatics and EngineeringThe University of Electro-Communications 13133 Tokyo 1828585 Japan; Center for Neuroscience and Biomedical EngineeringThe University of Electro-Communications 13133 Tokyo 1828585 Japan; Beijing Advanced Innovation Center for Intelligent Robots and Systems 554485 Beijing 100081 China; Graduate School of Informatics and EngineeringThe University of Electro-Communications 13133 Tokyo 1828585 Japan; Center for Neuroscience and Biomedical EngineeringThe University of Electro-Communications 13133 Tokyo 1828585 Japan; Beijing Advanced Innovation Center for Intelligent Robots and Systems 554485 Beijing 100081 China

**Keywords:** Control system, EMG shoulder disarticulation prosthesis, man-machine interface, mixed reality, wearable robot

## Abstract

*Goal*: The development of a control system for an electromyographic shoulder disarticulation (EMG-SD) prosthesis to rapidly achieve a task with a reduction in the operational failure of the user. *Methods*: The motion planning of an EMG-SD prosthesis was automated using measured visual information through a mixed reality device. The detection of an object to be grasped and motion execution depended on the EMG of the user, which gives voluntary controllability and makes the system semi-automated. Two evaluation experiments with reaching and reach-to-grasp movements were conducted to compare the performance of the conventional system when operated using only visual feedback control of the user. *Results*: The proposed system can more rapidly and accurately achieve reaching movements (32% faster) and more accurate (69%) reach-to-grasp movements than a conventional system. *Conclusions*: The proposed control system achieves a high task performance with a reduction in the operational failure of an EMG-SD prosthesis user.

## I. Introduction

A shoulder disarticulation prosthesis is used to reconstruct the function and appearance of the arm in people who have lost an upper limb, particularly a shoulder, owing to an accident or congenital problem. Because a body-powered shoulder disarticulation prosthesis requires alternative physical movements of the user, an electric shoulder disarticulation prosthesis driven by actuators is an area of focus [1]. Among them, an electromyographic shoulder disarticulation (EMG-SD) prosthesis, which uses the user's myoelectricity as a control input, can be operated intuitively using biological signals [2]–[6]. Compared with an EMG prosthesis for forearm amputees, an EMG-SD prosthesis has many movable body parts, that is, many degrees of freedom (DoFs) in terms of control. Therefore, both the hardware and control methods of an EMG-SD prosthesis face numerous challenges.

The well-known EMG-SD prostheses, Luke Arm [3] and Proto2 [4], developed by DARPA, allow for EMG-based manipulations of the arm with many degrees of freedom, which requires targeted muscle reinnervation (TMR) surgery [7]–[11]. TMR surgery is a procedure that reconnects the peripheral nerve at an amputation stump to the remaining muscles of the trunk. Because the EMG information related to the arm and hand can be measured at the trunk of the body, the arm movements of an EMG-SD prosthesis can be controlled by a pattern recognition-based control method [12]–[17]. However, the need for surgery and the time required for postoperative rehabilitation may place a heavy burden on EMG-SD prosthesis users [18], [19].

Our research group developed a simple EMG-SD prosthesis that can manipulate a two-degree-of-freedom (2-DoF) robotic arm and a 2-DoF robotic hand using only surface EMG and pattern recognition techniques [6]. We demonstrated that the developed simple EMG-SD prosthesis can grasp and move an object in three-dimensional (3D) space without long-term training by utilizing the user's body movements. However, a simple EMG-SD prosthesis is operated based only on the user's visual feedback control. Therefore, it is difficult to conduct a task rapidly owing to the slow visual feedback control of the EMG-SD prosthesis. Similar results have been reported in the EMG prosthesis system for forearm amputees [20].

In general, the visual feedback loop in the human central nervous system has a time delay of several 100 ms [21], [22]. However, the time delay is thought to be compensated by building internal models of the body in the brain [23], [24]. It has also been reported that visual feedback is more important in reaching movements before a movement than during a movement [25]. Therefore, it is suggested that humans perform rapid reaching movements in a feed-forward control manner by determining the motor conditions from visual information prior to the onset of movements and planning the reaching movements using internal models. Similarly, the control of the EMG-SD prosthesis will be separated from the slow visual feedback control loop of the user by detecting the motor condition and planning the motion through visual information obtained by the control system itself. In our conventional system [6], the user always relies on his/her own visual information to recognize the object to be grasped, the state of the robotic arm, and the motion of the robotic arm being performed. Therefore, when the user performs the reaching movement with the conventional EMG-SD prosthesis system, he/she must always visually check whether the robotic hand has reached the object. In addition, when the user performs correcting movements, he/she has to re-plan and re-execute the motion using the human slow visual feedback. To improve the control of such a slow EMG-SD prosthesis system, the visual information of the external environment needs to be acquired and reflected in motion planning.

The objective of this study is to develop a control system for an EMG-SD prosthesis that can accomplish a task rapidly while reducing the operational failure. To achieve this objective, we developed a semi-automated control system for reaching movements in an EMG-SD prosthesis using a mixed reality (MR) device [26]. The MR device measures the external environment with a camera and a depth sensor attached on a head-mounted display and can display information considering the shape of the external environment to the wearer. Here, “semi-automated” means that the EMG-SD prosthesis system does not perform all movements automatically, allowing a voluntary controllability to give the user a sense of agency [27], [28]. Several earlier studies have reported on how to operate a robot arm using visual information [29]–[32]. However, unlike these studies, this study challenges the semi-automated control of reaching movements in the wearable EMG-SD prosthesis with voluntary controllability depending on the intuitive biological signal, that is, an EMG.

## II. Materials and Methods

### A. System Overview

In this study, we propose a semi-automated control system for reaching movements in an EMG-SD prosthesis using an MR device. The control system acquires the visual information by using the MR device instead of the user and utilizes it as one of the control inputs. By using the acquired visual information, the proposed control system can eliminate the slow visual feedback loop for the EMG-SD prosthesis user and can achieve a fast pseudo-feedforward control to reduce the operational failure of the user. In addition, the proposed system automatically plans the motion of the EMG-SD prosthesis arm based on the visual information acquired to simplify the operational procedures.

**Fig. 1.**
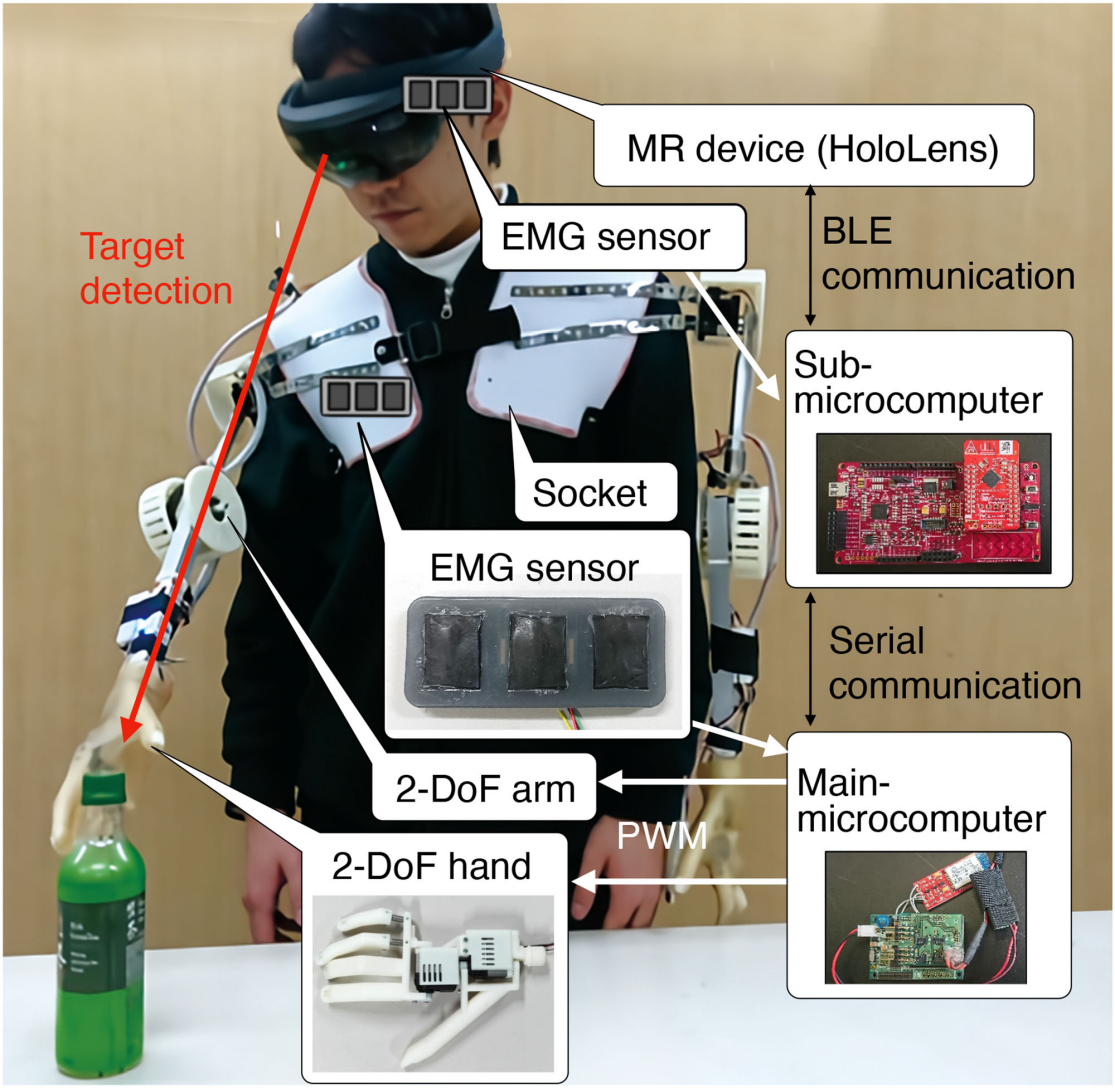
Overview of the proposed control system for an electromyographic shoulder disarticulation prosthesis. The user gazes at an object to be grasped and then inputs a user interface operational EMG signal measured from his/her own temple. The MR device measures the linear distance to the object to be grasped and the user's head angle as visual information. The measured visual information is sent to a sub-microcomputer via BLE communication. The sub-microcomputer calculates the target shoulder angle and sends a command to a main-microcomputer via serial communication. When the user inputs a motion operational EMG signal measured from his/her own trunk, the main-microcomputer actuates a robotic arm and achieves the reaching movement.

Fig. 1 shows an overview of the proposed control system for the EMG-SD prosthesis. As a control target, we used a simple EMG-SD prosthesis developed by our research group [6]. HoloLens (Microsoft, USA) was used as the MR device to measure the visual information of the external environment. The main-microcomputer (SH72544R, Renesas Electronics Corp., Japan) controls the 2-DoF arm and the 2-DoF hand by measuring the EMG from the trunk of the user as control inputs. The visual information acquired from the HoloLens and the EMG from the temple of the user are sent to a sub-microcomputer (PSoC4, Cypress Semiconductor Co., USA) for target detection and motion planning. In this study, the HoloLens device acquires the linear distance to the object to be grasped as visual information. Moreover, the rotation of the user's head is captured by the inertial measurement unit (IMU). Thus, the MR device can detect the position of the object to be grasped in a 3D user coordinate system. The target trajectory determined from the acquired visual information is sent from the sub-microcomputer to the main-microcomputer to control the robotic hand and robotic arm. The proposed system calculates the target shoulder angle as the target trajectory. Then, the robotic shoulder is moved to the target shoulder angle while the control EMG signal acquired from the user's trunk is input. While the control EMG signal is not input, the movement of the robotic arm is stopped. Such an EMG-triggered control provides the user voluntary controllability. In the following, we describe the components of the system in detail.

The hardware of the simple EMG-SD prosthesis consists of three components: a socket, a robotic arm, and a robotic hand. The socket fixes the robotic arm to the user's body. The material of the socket is a thermoplastic resin. The socket is designed to be mounted on an average adult male torso. The joints of the robotic arm are moved by a direct-drive with servo motors (KRS6003RHV, Kondo Kagaku Co., Ltd., Japan) attached to the base of each shoulder and elbow, and generate flexion and extension movements on the sagittal plane. The length of the upper arm is 225 mm, and the forearm length is 255 mm. The 2-DoF robotic hand is based on an EMG prosthetic hand developed by our research group [33], [34]. The skeleton of the hand is made of a 3D-printed resin. Two servo motors (2BBMG, GWS Co., Ltd., China) are placed at the base of the thumb and four fingers, respectively. Moreover, the robotic hand is covered with an elastomeric glove [35] to give the hand an appearance similar to a healthy human hand and improve the gripping performance owing to friction. The total weight of the hardware is approximately 1.3 kg (Socket, 350 g; Robotic arm, 750 g; Robotic hand and glove, 200 g).

The EMG sensor consists of three components: a dry electrode, an amplifier, and a case. As shown in Figure 1, the EMG sensor is equipped with two exploration electrodes at both ends and a central reference electrode in a silicone case, with a built-in amplifier (AD620, Analog Devices Inc., USA). The two-layered conductive silicone electrodes developed by our research group [36] are used for the electrodes to realize a stable EMG measurement without a gel. The entire sensor is covered with silicone, which is waterproof and robust for measuring the EMG even when the user is sweating.

### B. Control Method of Proposed System

**Fig. 2.**
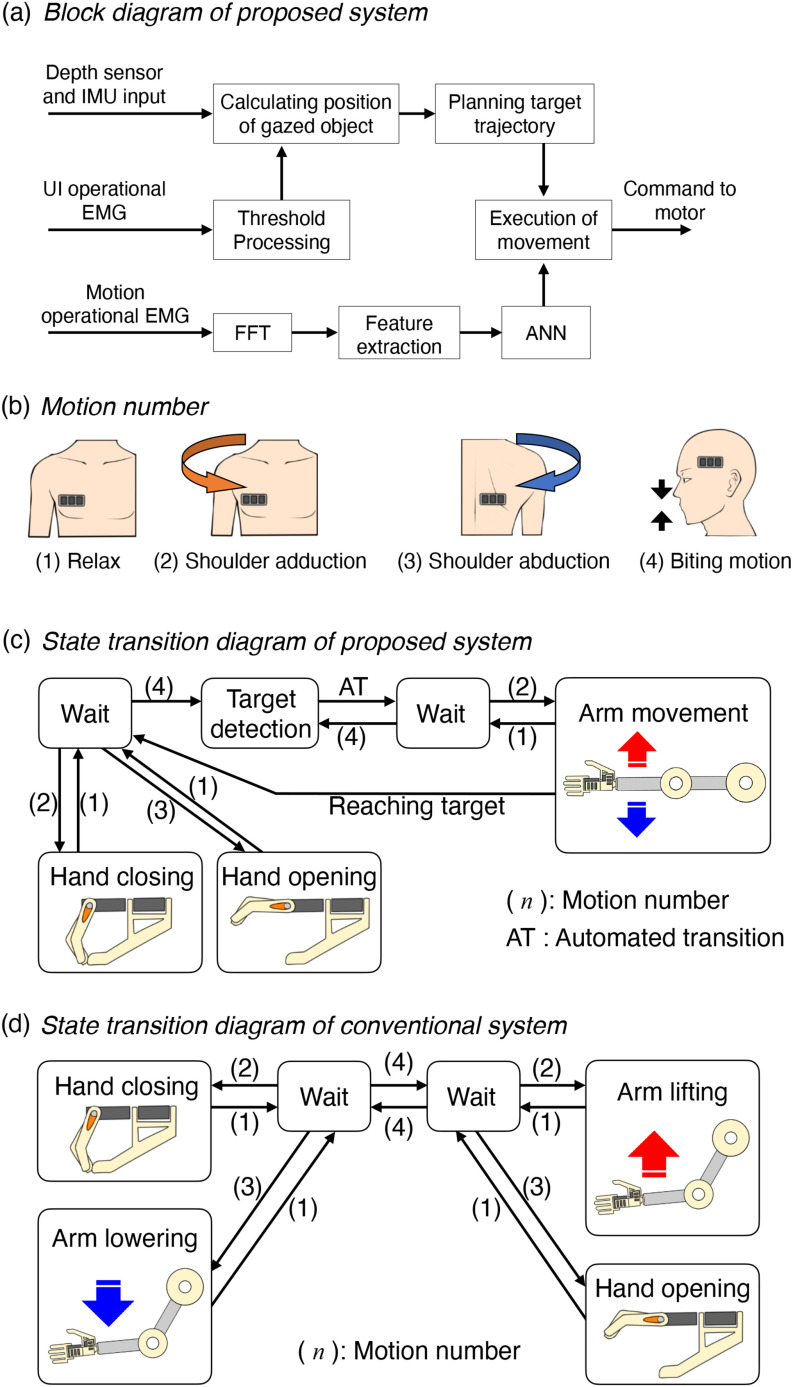
Control method of the proposed system. (a) Block diagram of the proposed system. The depth sensor and inertia measurement unit measure the linear distance to the object to be grasped and the user's head angle, respectively. When the UI operational EMG is input, the position of the object to be grasped is calculated. Then, the target trajectory of the robotic arm is planned. The fast Fourier transform is applied to the measured motion operational EMG. Feature values are extracted from the transformed EMG data and input to the artificial neural network. The movement of the robotic arm is performed depending on the output of the ANN. (b) Numbered motions of the user. (c) State transition diagram of the proposed system. The numbers in the diagram correspond to the numbered motions of the user. Initially, the system is in the state of the upper left “wait” block. The motions 2 and 3 of the user correspond to the movements of the robotic hand opening and closing, respectively. When the motion 4 of the user is performed, the target object to be grasped is detected. Then, the motion 2 of the user corresponds to the movement of the robotic arm. Finally, the system returns to the initial state. (d) State transition diagram of the conventional system. Initially, the system is in the state of the right “wait” block. In the right “wait” block, the motions 2 and 3 of the user correspond to the movements of the robotic arm lifting and hand opening, respectively. When the motion 4 of the user is performed in the right “wait” block, the state translates to the left “wait” block, and vice versa. In the left “wait” block, the motions 2 and 3 of the user correspond to the movements of the robotic hand closing and arm lowering, respectively.

In this subsection, we describe the control method of the proposed system. In the system, two main algorithms are running: The first is an algorithm that detects the target of the reaching movement from the visual information obtained by the HoloLens and plans the movement of the robotic arm. The second is an algorithm that estimates the motor intention of the user based on the EMG information measured from the body trunk. The proposed system combines these two algorithms to operate the EMG-SD prosthesis in the manner shown in Fig. 2.

By combining the above two algorithms, the reaching movement of the EMG-SD prosthesis is controlled semi-automatically, as shown in Figs. 2a and 2c. The proposed control method can be briefly described as follows: Initially, the proposed system is in the hand operational mode, in which the shoulder adduction and abduction of the user correspond to the robotic hand closing and opening, respectively. When the user gazes at the object to be grasped and inputs the user interface (UI) operational EMG with a biting motion, the target is detected, the motion of the arm is planned, and the proposed system shifts to the arm operational mode. When the shoulder adduction motion is input in the arm operational mode, the pre-planned arm motion is executed. When the user rests during the robotic arm movement, the motion of the EMG-SD prosthesis is stopped. This pause function makes a voluntary operation of the user possible and is the reason for the “semi-automatic” control of the reaching movement. When the tip of the arm reaches the target, the proposed system automatically shifts to the hand operational mode. After grasping the target object, the user gazes at another target and executes the arm motion again to achieve the grasping and moving of the object. The details of the two algorithms are given below.

The visual information measured by the HoloLens is the linear distance to the target object to be grasped and the head angle of the user. The linear distance and head angle were measured by the depth sensor and inertial measurement unit mounted on the HoloLens, respectively. The proposed system can calculate the position of the target object in three-dimensional space by using these measured quantities. Specifically, the target position is projected onto the sagittal plane using the measured head angle of the user. Then, the system can acquire the target position in the user coordinate system with the center of the user's body as the origin. The target trajectory is then planned such that the EMG-SD prosthesis hand can reach the target object in the user coordinate system. To simplify the problem, we assume that the origin of the EMG-SD prosthesis (the center of rotation of the shoulder joint) is fixed and given in the user coordinate system, and only the shoulder joint is moved with the elbow extended. The proposed system detects the relative position of the target object at a time when the EMG information for the UI operation (described in a later paragraph) is generated by the user. In this study, the UI operational EMG signal is activated by the biting motion of the user. The information of the detected target is then sent to the sub-microcomputer. The sub-microcomputer plans the target trajectory and sends the target shoulder angle to the main-microcomputer. In this study, we considered only the shoulder movement; therefore, the main-microcomputer drives the motor of the shoulder joint according to the target angle.

In the proposed system, two types of EMG information are used as a control input: The first is the EMG information for the UI operation in the HoloLens, and the second is the EMG information for the execution of the motion of the robotic arm and hand. The UI operational EMG is measured using a sub-microcomputer with a sampling frequency of 2000 Hz and is full-wave rectified. When the average value across 40 data points (20 ms) exceeds the threshold, the sub-microcomputer interprets that the UI operational EMG has been input. In this study, an EMG signal from the temple of the user, which is activated by a biting motion, is used as the UI operational EMG. The user can directly view the target object to be grasped via the see-through display of the HoloLens. The application for visual feedback runs at 60 FPS in HoloLens. When the user inputs the UI operational EMG, the HoloLens application provides visual feedback to the user in the form of a pink rectangular image whose center position is the location of the target object. In the proposed system, the user can view and target the object to be grasped in real time.

The signal processing method of the motion operational EMG is the same as that of the conventional system [6]. The motion operational EMGs were measured by the main-microcomputer with a sampling frequency of 2000 Hz, filtered using a second-order Butterworth high-pass filter with a cutoff frequency of 50 Hz. The fast Fourier transform was applied to the filtered 256-point EMG data every 12.8 ms. Then, the average values of each power spectrum in the eight frequency domains (39.1–70.3 Hz, 54.7–85.9 Hz, 70.3–101.6 Hz, 93.8–125.0 Hz, 125–156.3 Hz, 171.9–203.1 Hz, 234.4–265.6 Hz, 312.5–343.8 Hz) were used as the input of the three-layered artificial neural network (ANN) [6], [37]. The number of output layers of the ANN corresponds to the number of motion patterns. The control system considers three movements of the user: resting, shoulder adduction, and abduction. By using such a pattern recognition technique, noise is treated as non-discriminatory, which reduces the malfunctions of the EMG-SD prosthesis compared to the threshold-based control. In this study, the EMG sensors for motion operation were attached to the pectoralis major and broad back muscles, respectively. The correspondence between the motion of the user and the motion label of the EMG-SD prosthesis is mapped through the UI on the HoloLens. Therefore, the user can conduct the learning process by using only the UI operational EMG without an assistant.

### C. Control Method of Conventional System

Fig. 2d shows the conventional control method proposed in our previous study [6]. The location of the EMG sensor and the EMG information processing method using the ANN were the same as those used by the proposed method. A biting motion is assigned to the switching function between the two motion modes. In the first mode, the shoulder adductions and abductions of the user correspond to robotic hand closing and robotic arm lowering, respectively. In the second mode, the shoulder adductions and abductions of the user correspond to robotic arm lifting and robotic hand opening, respectively. The status of the motion mode is fed back to the user using LEDs attached to the robotic forearm. In a conventional system, the flexion and extension of the shoulder movements of the user correspond to the flexion and extension movements of the robotic hand and robotic arm, respectively. A switching function is required between the hand opening and closing movements to prevent the grasped object from being dropped. Unlike the proposed system, visual feedback control at the user end is used to control the switching and perception of the motion mode and execute the movements.

### D. Evaluation Experiments

Two evaluation experiments were conducted to confirm the effectiveness of the proposed system. We conducted experiments on reaching and reach-to-grasp movements to evaluate the performance of the semi-automated reaching movement method and the performance of the entire system, respectively. For a comparison with the proposed system, a conventional EMG-SD prosthesis control system depending on the visual feedback control of the user was applied [6]. The control method of the conventional system is shown in the supplementary materials (Fig. S1).

Seven healthy subjects (6 males and 1 female, mean age 23.7±1.9 years) participated in the experiments. The experiments were approved by the Ethics Committee of the University of Electro-Communications (No. 100006(5)), and informed consent was obtained from the subjects in writing. Before the experiments, the subjects were given a lecture on how to operate each system and practiced for a few minutes. During all experiments, the proposed system and the conventional system were tested in order. Four speeds of the shoulder motor (normal, 0.32 rad/s, 0.57 rad/s, 0.85 rad/s, and fastest, 1.06 rad/s) were used to evaluate the change in performance as the speed increased. The normal speed was heuristically determined in conventional systems to easily operate the robotic arm and hand. In all experiments, the task was performed during five trials for each speed condition.

**Fig. 3.**
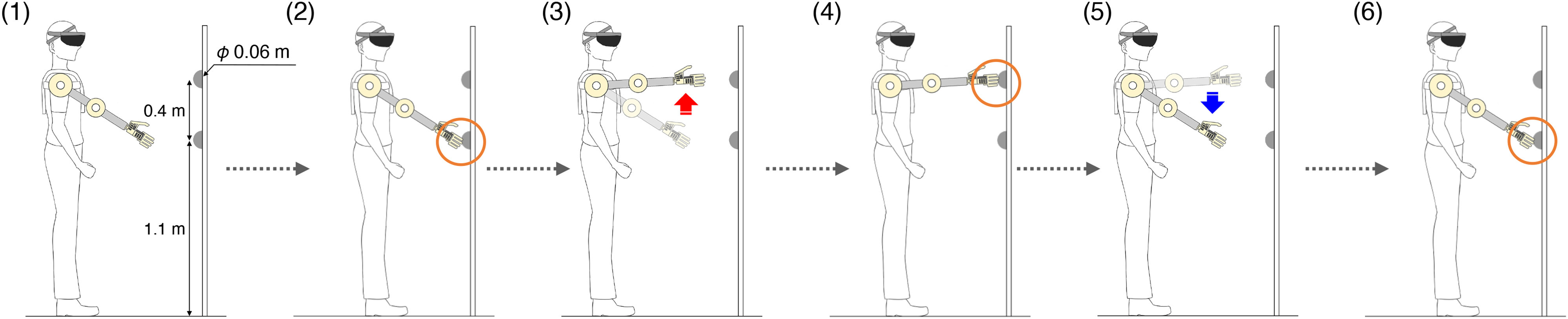
Experimental procedure in the experiment evaluating reaching movements. (1) The subject stands in front of the wall. (2) The time when at which the robotic hand touched touches the lower target is set as the start of the measurement. (3) The subject moves the robotic arm to the upper target. (4) The subject touches the upper target. (5) The subject then moves the robotic arm to the lower target again. (6) Finally, the subject touches the lower target.

The experimental procedure for evaluating the reaching movements is shown in Fig. 3, and a movie showing the experiments is provided in Supplementary Movie 1. In the experiment evaluating the reaching movements, we evaluated the time taken to reciprocally move the robotic hand between two targets with a diameter of 6 cm attached to a vertical wall. In the proposed system, subjects stood in front of a wall upon which a target was placed and the HoloLens was calibrated (user coordinate system). In the calibration process, the HoloLens system was calibrated with the head angle set to zero when the user was gazing in the horizontal direction. After the start of the experiment, the subject stood in front of the wall, moved the tip of the robotic hand to a position where it could touch the lower target, and waited (Fig. 3(1)). The time when the robotic hand touched the lower target was set as the start of the measurement (Fig. 3(2)). After confirming that the subject touched the lower target, the subject moved the robotic arm to the upper target (Fig. 3(3)) and touched the upper target (Fig. 3(4)). The subject then moved the robotic arm to the lower target again (Fig. 3(5)) and touched it (Fig. 3(6)). The time taken for the above movement was measured manually using a stopwatch. If the subject did not touch the target, the trial was conducted again.

**Fig. 4.**
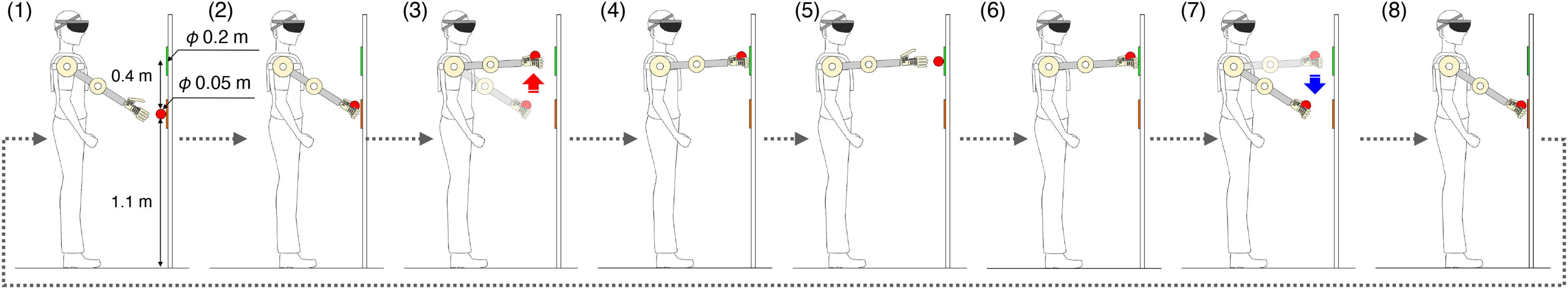
Experimental procedure in the experiment evaluating reach-to-grasp movements. (1) The subject stands in front of the wall. (2) The subject grasps the ball placed at the lower target and takes it from the target. (3) While holding the ball, the robotic arm is moved to the upper target. (4) The ball is attached to the upper target. (5) The robotic hand is opened and the hand is released from the ball. (6) The subject grasps the ball placed at the upper target again. (7) The subject lowers the robotic arm to the lower target. (8) The subject attaches the ball to the lower target. The robotic hand is released from the ball. The subjects repeat the above procedures for 1 min.

The experimental procedure for evaluating the reach-to-grasp movements is shown in Fig. 4, and a movie of the experiments is provided as Supplementary Movie 2. In the experiment conducted to evaluate the reach-to-grasp movements, we evaluated the number of times the ball was reciprocally carried between two targets during a 1 min period [6]. Two 20-cm diameter hook-and-loop fastener targets were placed on a vertical wall. A soft cloth ball with a diameter of approximately 5 cm was attached to the lower target. Before the experiments, using the proposed system, the subjects stood in front of a wall upon which a target was placed and the HoloLens (user coordinate system) was calibrated. After the start of the experiment, the subject stood in front of the wall, moved the tip of the robotic hand to a position where it could touch the ball placed at the lower target, and waited (Fig. 4(1)). After the experimenter signaled the start of the measurement, the subject grasped the ball placed at the lower target and took it from the target (Fig. 4(2)). Then, while holding the ball, the robotic arm was moved to the upper target (Fig. 4(3)), and the ball was attached to it (Fig. 4(4)). The hand was then opened and the hand was released from the ball (Fig. 4(5)). The subject again grasped the ball placed at the upper target (Fig. 4(6)), lowered the robotic arm to the lower target (Fig. 4(7)), attached the ball to it (Fig. 4(8)), and released the robotic hand from the ball (Fig. 4(1)). The subjects repeated the above procedures for 1 min. The experimenter recorded the number of times the ball moved from one target to another to determine the number of successes. If the ball dropped after being attached to the target, the experimenter picked the ball up and attached it to the target. If the ball dropped during the arm movement, it was attached by the experimenter to the same target to which it was attached at the start of the arm movement. If the ball dropped after touching the target, it was counted as a success.

### E. Data Analysis

We recorded the time taken to conduct the task during the experiment evaluating the reaching movements, and the number of successes during the experiment evaluating reach-to-grasp movements. For each subject, the mean values of the evaluation index under each speed condition were calculated. We also calculated the changes in both mean indices between the normal and fastest conditions. The number of overshoots and undershoots to the target was also recorded during both evaluation experiments. The overshoot and undershoot were defined as when the robotic hand passed the target and a corrective or compensatory movement was conducted by the EMG-SD prosthesis or trunk of the user, and when the robotic hand did not reach the target and a corrective or compensatory movement was conducted by the EMG-SD prosthesis or trunk, respectively. The mean values of the sum of the number of over- and undershoots throughout the trials and the changes between the normal and fastest conditions were also calculated. We also recorded the number of operational failures in the experiment evaluating reach-to-grasp movements. An operational failure was defined as the arm operating in a state by which the hand should be operated, and vice versa.

### F. Statistical Analysis

A two-way repeated-measures analysis of variance (ANOVA) was performed to examine the effect of the system (proposed or conventional) and the speed conditions on the time taken to conduct the task in the experiment evaluating reaching movements, the average sum of the number of over- and undershoots in both evaluation experiments, and the number of successes in the experiment evaluating reach-to-grasp movements (system conditions: 2 × angular velocity conditions: 4, *α* = 0.05). A paired *t*-test (*α* = 0.05) was performed to compare the changes in the time taken to conduct the task, the change in the average sum of the number of overshoots and undershoots, and the number of successes in the proposed and conventional systems. The correlation coefficients were calculated to evaluate quantitatively the change in the time taken to conduct the task and the number of successes, depending on the change in the speed of the robotic shoulder.

## III. Results

### A. Evaluation of Reaching Movements

**Fig. 5.**
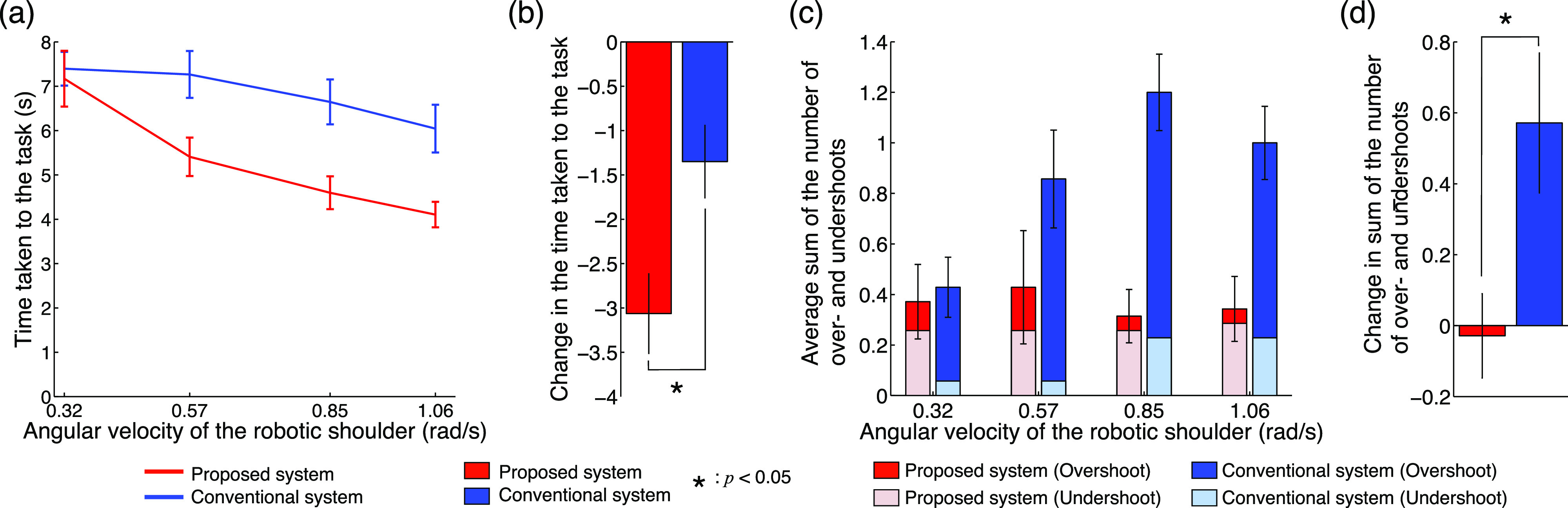
Results of the experiments evaluating reaching movements. (a) Time taken to conduct the task for each speed condition. The vertical and horizontal axes denote the time taken to conduct the task and the angular velocity of the robotic shoulder, respectively. The red and blue lines indicate the results of the proposed and conventional systems, respectively. The error bars denote the standard errors across subjects. (b) Change in the time taken to conduct the task between the normal and fastest conditions. The red and blue bars indicate the results of the proposed and conventional systems, respectively. The asterisk indicates a significant difference. (c) Average sum of the number of over- and undershoots in each speed condition. The vertical and horizontal axes denote the average sum of the number of over- and undershoots and the angular velocity of the robotic shoulder, respectively. The red, pink, blue, and aqua bars denote the over- and undershoots of the proposed system and those of the conventional system, respectively. (d) Change in the average sum of the number of over- and undershoots between the normal and fastest conditions.

Fig. 5 shows the results of the experiment evaluating the reaching movements. The time taken to complete the task decreased as the robotic shoulder speed increased in both systems (Fig. 5a). Notably, the proposed system decreased the time (average 32% decrease from normal to fastest conditions). In the fastest condition, all the subjects required a shorter time with the proposed system than with the conventional system. The length of time taken to complete the task from the normal to the fastest conditions when applying the proposed system was approximately 1.5 s faster than that of a conventional system (Fig. 5b). The average sum of the number of over- and undershoots increased in the conventional system, whereas the proposed system changed slightly with an increase in the speed of the robotic shoulder (Fig. 5c). The difference from the normal to the fastest conditions also remained mostly unchanged in the proposed system, whereas the conventional system increased by an average of approximately 0.6, that is, the number of over- and undershoots increased in three of the five trials (Fig. 5d). These results clearly demonstrate that the proposed system can perform reaching movements with a higher accuracy and speed than the conventional system.

The statistical analysis supported the above results. A two-way ANOVA showed that there was no significant interaction (*p* = 0.183 > 0.05, *F* value, and *F*_(3, 48)_ = 1.69), with significant effects from the speed (*p* = 1.64 × 10^-4^ < 0.05 and *F*_(3, 48)_ = 8.21) and system (*p* = 3.58×10^-5^ < 0.05, *F*_(1, 48)_ = 20.76) conditions for the time taken to complete the task (Fig. 5a). Therefore, the time required for the task was significantly shorter with the proposed system than with the conventional system. A correlation analysis revealed a significant negative correlation between the time taken to complete the task depending on the increase in the speed of the robotic shoulder in both systems (proposed system, *r* = -0.966 and *p* = 0.034 < 0.05; conventional system, *r* = -0.965 and *p* = 0.035 < 0.05). A paired *t*-test indicated that the difference from the normal to the fastest conditions was significantly greater for the proposed system (*p* = 0.019 < 0.05, *t* value: *t*_(6)_ = -3.18, Fig. 5b). A two-way ANOVA showed that there was no significant interaction (*p* = 0.066 > 0.05, *F*_(3, 48)_ = 2.56), no significant effect from the speed conditions (*p* = 0.139 > 0.05 and *F*_(3, 48)_ = 1.92), and a significant effect from the system conditions (*p* = 3.21×10^-5^ < 0.05 and *F*_(1, 48)_ = 21. 06) for the sum of the number of over- and undershoots (Fig. 5c). Therefore, the sum of the number of over- and undershoots was significantly lower across all the speed conditions in the proposed system. A paired *t*-test showed that the difference from the normal to the highest conditions was significantly lower for the proposed method (*p* = 0.046 < 0.05 and *t*_(6)_ = -2.51, as indicated in Fig. 5d).

The above results show that the performance of the task was significantly improved by the semi-automated control of the reaching movements, particularly for rapid movements. Specifically, the significance of the proposed system was demonstrated even for simple tasks such as simple reaching movements, and the operational failure of the user will be reduced for the proposed system.

### B. Evaluation of Reach-to-Grasp Movements

**Fig. 6.**
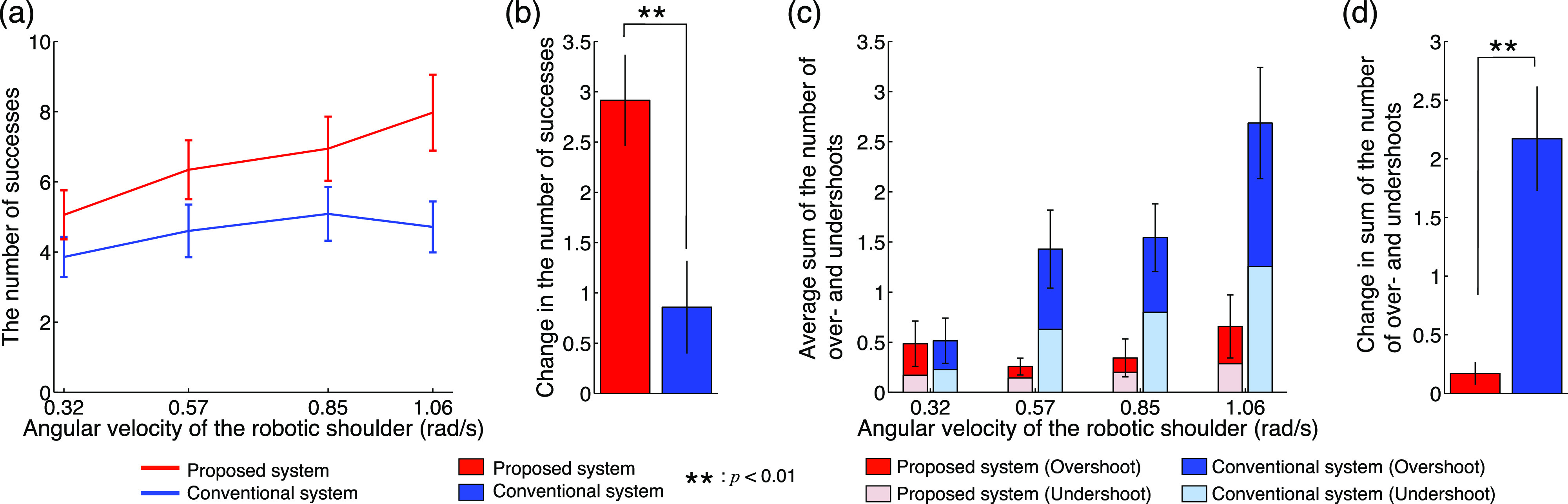
Results of the experiments evaluating reach-to-grasp movements. (a) Number of successes under each speed condition. The vertical and horizontal axes denote the number of successes and the angular velocity of the robotic shoulder, respectively. The red and blue lines indicate the results of the proposed and conventional systems, respectively. The error bars denote the standard errors across subjects. (b) Change in the number of successes from the normal to fastest conditions. The red and blue bars indicate the results of the proposed and conventional systems, respectively. The asterisk indicates a significant difference. (c) Average sum of the number of over- and undershoots under each speed condition. The vertical and horizontal axes denote the average sum of the number of over- and undershoots and the angular velocity of the robotic shoulder, respectively. The red, pink, blue, and aqua bars denote the over- and undershoots of the proposed system and those of the conventional system, respectively. (d) Change in the average sum of the number of over- and undershoots from the normal to fastest conditions.

**Fig. 7.**
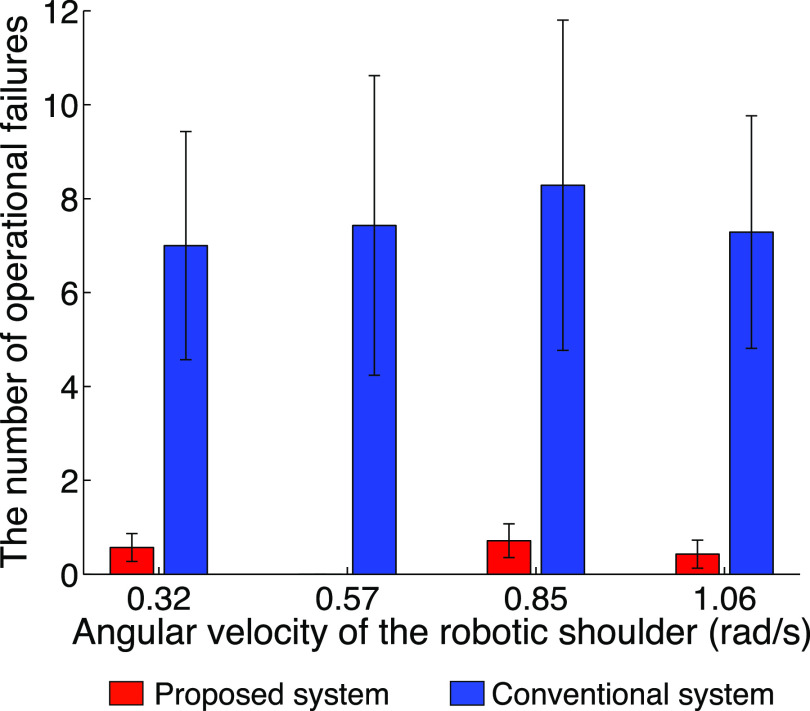
Number of operational failures under each speed condition. The vertical and horizontal axes denote the number of operational failures and the angular velocity of the robotic shoulder, respectively. The red and blue bars indicate the results of the proposed and conventional systems, respectively. The error bars denote the standard errors across subjects.

Fig. 6 shows the results of the experiment evaluating the reach-to-grasp movements. The number of successes of the proposed system linearly increased depending on the increase in the speed of the robotic shoulder, whereas a plateau appeared in the conventional system (Fig. 6a). Moreover, the proposed system demonstrated a greater number of successes overall (average increase of 69% from the normal to the fastest conditions). In the fastest condition, all the subjects showed a greater number of successes with the proposed system than with the conventional system. The change in the number of successes from the normal to the fastest conditions was larger in the proposed system than that in the conventional system, i.e., specifically, an average of more than twice that of the conventional system was achieved (Fig. 6b). The average sum of the number of over- and undershoots linearly increased in the conventional system, whereas the proposed system changed little depending on the increase in the speed of the robotic shoulder (Fig. 6c). The difference from the normal to the fastest conditions in the proposed system did not change significantly, whereas the conventional system showed a twofold increase on average (Fig. 6d). The number of operational failures is shown in Fig. 7. Almost no operational failures were observed for the proposed system, whereas an average of approximately seven operational failures was found across all speed conditions for the conventional system. These results clearly demonstrate that the proposed system reduces the number of operational failures and enables rapid and accurate reach-to-grasp movements compared with a conventional system.

The statistical analysis supported the above results. A two-way ANOVA showed that there was no significant interaction (*p* = 0.624 > 0.05 and *F*_(3, 48)_ = 0.59), no significant effect from the speed conditions (*p* = 0.114 > 0.05 and *F*_(3, 48)_ = 2.09), and a significant effect from the system conditions (*p* = 9.26 × 10^-4^ < 0.05 and *F*_(1, 48)_ = 12.47) on the number of successes (Fig. 6a). Therefore, the number of successes was significantly higher for the proposed system. A correlation analysis showed that the number of successes was significantly and positively correlated with the increase in speed for the proposed system (*r* = 0.987 and *p* = 0.013 < 0.05). By contrast, the conventional system did not show a significant correlation (*r* = 0.79 and *p* = 0.206 > 0.05). A paired *t*-test indicated that the difference from the normal to the fastest conditions was significantly greater for the proposed system (*p* = 0.009 < 0.05 and *t*_(6)_ = 3.80, as shown in Fig. 6b). A two-way ANOVA showed that there was a significant interaction (*p* = 0.028 < 0.05 and *F*_(3, 48)_ = 3.31), a significant effect from the speed conditions (*p* = 0.006 < 0.05 and *F*_(3, 48)_ = 4.77), and a significant effect from the system conditions (*p*=1.11×10^-5^ < 0.05 and *F*_(1, 48)_ = 24.07) for the average sum of the number of over- and undershoots (Fig. 6c). Therefore, the sum of the number of over- and undershoots was significantly lower in the proposed system across all the speed conditions. A paired *t*-test indicated that the difference from the normal to the fastest conditions was significantly lower in the proposed system (*p* = 0.002 and *t*_(6)_ = -5.26, as indicated in Fig. 6d). A two-way ANOVA showed that there was no significant interaction (*p* = 0.992 > 0.05 and *F*_(3, 48)_ = 0.03), no significant effect from the speed conditions (*p* = 0.981 > 0.05 and *F*_(3, 48)_ = 0.06), and a significant effect from the system conditions (*p* = 1.65×10^-5^ < 0.05 and *F*_(1, 48)_ = 22.93) on the number of operational failures. Therefore, the number of operational failures was significantly lower for the proposed system across all speed conditions.

The above results show that the performance of the RTG movements can also be improved by semi-automated control for the reaching movements. In a conventional system, the subjects had to control the rapid movement with a switching action by using only visual feedback control, particularly under the fastest condition, which would lead to a significant operational burden on the user and result in operational failures. By contrast, in the proposed system, because the arm movements, that is, the reaching movements, were under semi-automated control, rapid and accurate movements could be achieved. With the proposed system, the subjects can control the EMG-SD prosthesis by utilizing feedforward control rather than visual feedback control. Feedforward control will reduce the operational failure of the user.

## IV. Discussion

In this study, we proposed a semi-automated control system for reaching movements based on an MR device. As the results of two evaluation experiments indicate, our proposed system showed a higher performance than a conventional approach for both experiments, that is, the reaching movements were more rapidly and accurately achieved, and the reach-to-grasp movements were conducted more times and also more accurately achieved. These results suggest that the proposed system enables the user to achieve reaching movements through feedforward control, which reduces the operational failure of the user and makes the movements of EMG-SD prosthesis more rapid and accurate than those in a conventional system depending on only the visual feedback control of the user.

To simplify the problem, we assumed that the origin of the EMG-SD prosthesis (the center of rotation of the shoulder) was fixed and given in the user coordinate system, and only the shoulder joint was moved while the elbow was extended. In a case in which the origin of the EMG-SD prosthesis is moved, for example, the system can be calibrated by measuring the relative motion of the socket to the head. Alternatively, a calibration method based on image recognition of the EMG-SD prosthetic hand on the MR device is also possible. In the case of a simple 2-DoF EMG-SD prosthesis, an arbitrary hand trajectory can be easily achieved by solving the inverse kinematics problem. Even if the hardware system of the EMG-SD prosthesis is extended to a higher DoF robotic arm, the motion planning part of the system can be considered independently from the proposed system. Therefore, if the control system of the EMG-SD prosthesis plans a motion in a different framework, for example, using pseudo-inverse matrices for solving the inverse kinematics [38], the control system can detect the target and execute the movements in a proposed systemic manner. Moreover, applying a multi-DoF arm can extend the arm movements, which are limited to the sagittal plane in the present study, to movements within a 3D space. The extension to a multi-DoF hardware system is an important area of future research.

In this study, only the arm movements of the EMG-SD prosthesis were semi-automated, and voluntarily control of the hand movements remained through visual feedback of the user. In previous studies on EMG forearm prosthetic hands, a control system that uses image recognition techniques to achieve the optimal grasping shape for a target object has been proposed [39]–[44]. In the present study, it was difficult to automate the grasping process according to the shape of the grasped object because the hardware for both the arm and the hand had only 2-DoF. Our future work will aim to extend the proposed system to semi-automated control of the reach-to-grasp movements using a multi-DoF robotic arm and hand.

## V. Conclusion

In this study, a semi-automated control system for reaching movements was developed to reduce the operational failure of an EMG-SD prosthesis by applying an MR device. The proposed system was compared to a conventional system, under which all movements were operated through visual feedback control of the user, and based on two evaluation experiments. The experimental results showed that the proposed system can perform reaching movements more rapidly and accurately, and apply reach-to-grasp movements a larger number of times and with greater accuracy than the previous system. These results were particularly significant for rapid movements and suggest that the proposed system can enable users to conduct tasks through feedforward control rather than visual feedback control, thereby reducing the operational failure and improving the performance. The novelty of this study is that the proposed system could semi-automate the control of the EMG-SD prosthesis by combining the MR device with EMG-based control. The difference between this study and previous studies is that voluntary controllability by EMG-based control is retained. We conclude that the proposed system can achieve tasks with high performance while reducing the operational failure of the EMG-SD prosthesis user.

## Supplementary Material

The control method of the conventional system.

The movie of the experiments.

## References

[1] A. J. Metzger, A. W. Dromerick, R. J. Holley, and P. S. Lum, “Characterization of compensatory trunk movements during prosthetic upper limb reaching task,” Arc. Phys. Med. Rehab., vol. 93, no. 11, pp. 2029–2034, 2012. 10.1016/j.apmr.2012.03.011 22449551

[2] D. S. V. Bandara, R. A. R. C. Gopura, K. T. M. U. Hemapala, and K. Kiguchi, “Upper extremity prosthetics: Current status, challenges and future directions,” in Proc. Int. Symp. Artificial Life and Robotics, 2012, pp. 875–880.

[3] S. Adee, “Dean Kamen's” Luke Arm “prosthesis readies for clinical trials,” IEEE Spectr., vol. 2, no. 34, 2008.

[4] S. Adee, “A ‘Manhattan Project’ for the next generation of bionic arms,” IEEE Spectr., Mar. 2008.

[5] J. A. Leal-Naranjo, C. R. T. S. Miguel, M. Ceccarelli, and H. Rostro-Gonzalez, “Mechanical design and assessment of a low-cost 7-DOF prosthetic arm for shoulder disarticulation,” Appl. Bionics Biomech., vol. 4357602, 2018. 10.1155/2018/4357602 PMC6140102 30250502

[6] S. Kimizuka, Y. Tanaka, S. Togo, Y. Jiang, and H. Yokoi, “Development of a shoulder disarticulation prosthesis system intuitively controlled with the trunk surface electromyogram,” Front. Neurorobot.*,* vol. 15, 2020, Art. no. 542033. 10.3389/fnbot.2020.542033 PMC7658101 33192432

[7] T. A. Kuiken , “Targeted reinnervation for enhanced prosthetic arm function in a woman with a proximal amputation: A case study,” Lancet, vol. 369, pp. 371–380, 2007. 17276777 10.1016/S0140-6736(07)60193-7

[8] T. A. Kuiken , “Targeted muscle reinnervation for real-time myoelectric control of multifunction artificial arms,” JAMA, vol. 301, no. 6, pp. 619–628, 2009. 19211469 10.1001/jama.2009.116 PMC3036162

[9] J. M. Souza , “Targeted muscle reinnervation: A novel approach to postamputation neuroma pain,” Clin. Orthop. Relat. Res., vol. 472, no. 10, pp. 2984–2990, 2014. 24562875 10.1007/s11999-014-3528-7 PMC4160494

[10] T. A. Kuiken, A. K. Barlow, L. J. Hargrove, and G. A. Dumanian, “Targeted muscle reinnervation for the upper and lower extremity,” Tech. Orthop., vol. 32, no. 2, pp. 109–116, 2017. 28579692 10.1097/BTO.0000000000000194 PMC5448419

[11] G. A. Dumanian , “Targeted muscle reinnervation treats neuroma and phantom pain in major limb amputees: A randomized clinical trial,” Ann. Surg., vol. 270, no. 2, pp. 238–246, 2019. 30371518 10.1097/SLA.0000000000003088

[12] M. A. Oskoei and H. Hu, “Myoelectric control systems — A survey,” Biomed. Signal Process., vol. 2, pp. 275–294, 2007.

[13] E. Scheme and K. Englehart, “Electromyogram pattern recognition for control of powered upper-limb prostheses: State of the art and challenges for clinical use,” J. Rehabil. Res. Dev., vol. 48, pp. 643–659, 2011. 21938652 10.1682/jrrd.2010.09.0177

[14] P. Zhou , “Decoding a new neural-machine interface for control of artificial limbs,” J. Neurophysiol., vol. 98, no. 5, pp. 2974–2982, 2007. 17728391 10.1152/jn.00178.2007

[15] H. Huang, P. Zhou, G. Li, and T. A. Kuiken, “An analysis of EMG electrode configuration for targeted muscle reinnervation based neural machine interface,” IEEE Trans. Neur. Syst. Reh. Eng., vol. 16, no. 1, pp. 37–45, Feb. 2008. 10.1109/TNSRE.2007.910282 PMC4316368 18303804

[16] A. M. Simon, L. J. Hargrove, B. A. Lock, and T. A. Kuiken, “A decision-based velocity ramp for minimizing the effect of misclassifications during real-time pattern recognition control,” IEEE Trans. Biomed. Eng., vol. 58, no. 8, pp. 2360–2368, Aug. 2011. 10.1109/TBME.2011.2155063 PMC4269322 21592916

[17] L. J. Hargrove, L. A. Miller, L. Turner, and T. A. Kuiken, “Myoelectric pattern recognition outperforms direct control for transhumeral amputees with targeted muscle reinnervation: A randomized clinical trial,” Sci. Rep., vol. 7, 2017, Art. no. 13840. 10.1038/s41598-017-14386-w PMC5653840 29062019

[18] T. A. Kuiken, G. A. Dumanian, R. D. Lipschutz, L. A. Miller, and K. A. Stubblefield, “The use of targeted muscle reinnervation for improved myoelectric prosthesis control in a bilateral shoulder disarticulation amputee,” Prosthet. Orthot. Int., vol. 28, no. 3, pp. 245–253, 2004. 15658637 10.3109/03093640409167756

[19] J. E. Cheesborough, L. H. Smith, T. A. Kuiken, and G. A. Dumanian, “Targeted muscle reinnervation and advanced prosthetic arms,” Semin. Plast. Surg., vol. 29, no. 01, pp. 062–072, 2015. 10.1055/s-0035-1544166 PMC4317279 25685105

[20] J. V. V. Parr, S. J. Vine, N. R. Harrison, and G. Wood, “Examining the spatiotemporal disruption to gaze when using a myoelectric prosthetic hand,” J. Motor Behav., vol. 50, no. 4, pp. 416–425, 2018. 10.1080/00222895.2017.1363703 28925815

[21] L. G. Carlton, “Processing visual feedback information for movement control,” J. Exp. Psychol., vol. 7, no. 5, pp. 1019–1030, 1981. 10.1037//0096-1523.7.5.1019 6457106

[22] M. A. Khan , “Online versus offline processing of visual feedback in the control of movement amplitude,” Acta. Psychol., vol. 113, no. 1, pp. 83–97, 2003. 10.1016/s0001-6918(02)00156-7 12679045

[23] D. M. Wolpert, R. C. Miall, and M. Kawato, “Internal models in the cerebellum,” Trends Cogn. Sci., vol. 2, no. 9, pp. 338–347, 1998. 21227230 10.1016/s1364-6613(98)01221-2

[24] M. Kawato, “Internal models for motor control and trajectory planning,” Curr. Opin. Neurobiol., vol. 9, no. 6, pp. 718–727, 1999. 10607637 10.1016/s0959-4388(99)00028-8

[25] T. Fukui and T. Inui, “The effect of viewing the moving limb and target object during the early phase of movement on the online control of grasping,” Hum. Movement Sci., vol. 25, no. 3, pp. 349–371, 2006. 10.1016/j.humov.2006.02.002 16707178

[26] P. Milgram and F. Kishino, “A taxonomy of mixed reality visual displays,” IEICE Trans. Inf. Syst., vol. 77, no. 12, pp. 1321–1329, 1994.

[27] S. Gallagher, “Philosophical conceptions of the self: Implications for cognitive science,” Trends Cogn. Sci., vol. 4, pp. 14–21, 2000. 10637618 10.1016/s1364-6613(99)01417-5

[28] P. Haggard, “Sense of agency in the human brain,” Nat. Rev. Neurosci., vol. 18, pp. 196–207, 2017. 28251993 10.1038/nrn.2017.14

[29] I. Laffont , “Evaluation of a graphic interface to control a robotic grasping arm: A multicenter study,” Arch. Phys. Med. Rehab., vol. 90, no. 10, pp. 1740–1748, 2009. 10.1016/j.apmr.2009.05.009 19801065

[30] S. Dziemian, W. W. Abbott, and A. A. Faisal, “Gaze-based teleprosthetic enables intuitive continuous control of complex robot arm use: Writing and drawing,” in Proc. 6th IEEE Int. Conf. Biomed. Robot. Biomechatronics (BioRob), 2016, pp. 1277–1282.

[31] Y. Iwasaki and H. Iwata, “A face vector-the point instruction-type interface for manipulation of an extended body in dual-task situations,” in Proc. IEEE Int. Conf. Cyborg Bionic Syst., 2018, pp. 662–666.

[32] F. J. Chu, R. Xu, Z. Zhang, P. A. Vela, and M. Ghovanloo, “The helping hand: An assistive manipulation framework using augmented reality and tongue-drive interfaces,” in Proc. 40th Annu. Int. Conf. IEEE Eng. Med. Biol. Soc. (EMBC), 2018, pp. 2158–2161. 10.1109/EMBC.2018.8512668 30440831

[33] X. Jing, X. Yong, Y. Jiang, H. Yokoi, and R. Kato, “A low-degree of freedom EMG prosthetic hand with nails and springs to improve grasp ability,” in Proc. 7th Int. Conf. Biomed. Eng. Informat., 2014, pp. 562–567.

[34] S. Hoshigawa , “Structure design for a two-DoF myoelectric prosthetic hand to realize basic hand functions in ADLs,” in Proc. 37th Annu. Int. Conf. IEEE Eng. Med. Biol. Soc., 2015, pp. 4781–4784. 10.1109/EMBC.2015.7319463 26737363

[35] Y. Yabuki , “Development of new cosmetic gloves for myoelectric prosthetic hand using superelastic rubber,” Robot. Auton. Syst., vol. 111, pp. 31–43, 2019.

[36] S. Togo, Y. Murai, Y. Jiang, and H. Yokoi, “Development of an sEMG sensor composed of two-layered conductive silicone with different carbon concentrations,” Sci. Rep., vol. 9, 2019, Art. no. 13996. 10.1038/s41598-019-50112-4 PMC6768884 31570725

[37] R. Kato, H. Yokoi, and T. Arai, “Real-time learning method for adaptable motion-discrimination using surface EMG signal,” in Proc. IEEE/RSJ Int. Conf. Intell. Robots Syst., 2006, pp. 2127–2132.

[38] D. E. Whitney, “Resolved motion rate control of manipulators and human prostheses,” IEEE Trans. Human-Mach. Syst, vol. 10, no. 2, pp. 47–53, Jun. 1969.

[39] M. Markovic, S. Dosen, C. Cipriani, D. Popovic, and D. Farina, “Stereovision and augmented reality for closed-loop control of grasping in hand prostheses,” J. Neural Eng., vol. 11, 2014, Art. no. 046001. 10.1088/1741-2560/11/4/046001 24891493

[40] M. Markovic, S. Dosen, D. Popovic, B. Graimann, and D. Farina, “Sensor fusion and computer vision for context-aware control of a multi degree-of-freedom prosthesis,” J. Neural Eng., vol. 12, 2015, Art. no. 066022. 10.1088/1741-2560/12/6/066022 26529274

[41] F. Clemente, S. Dosen, L. Lonini, M. Markovic, D. Farina, and C. Cipriani, “Humans can integrate augmented reality feedback in their sensorimotor control of a robotic hand,” IEEE Trans. Human-Mach. Syst, vol. 47, no. 4, pp. 583–589, Aug. 2017.

[42] D. T. Andrade, A. Ishikawa, A. D. Munoz, and E. Rohmer, “A hybrid approach for the actuation of upper limb prostheses based on computer vision,” in Proc. Latin Amer. Robot. Symp. Braz. Symp. Robot., 2017, pp. 1–6.

[43] G. Ghazaei, A. Alameer, P. Degenaar, G. Morgan, and K. Nazarpour, “Deep learning-based artificial vision for grasp classification in myoelectric hands,” J. Neural Eng., vol. 14, 2017, Art. no. 036025. 10.1088/1741-2552/aa6802 28467317

[44] M. Markovic, H. Karnal, B. Graimann, D. Farina, and S. Dosen, “GLIMPSE: Google glass interface for sensory feedback in myoelectric hand prostheses,” J. Neural Eng., vol. 14, 2017, Art. no. 036007. 10.1088/1741-2552/aa620a 28355147

